# [RETRACTED ARTICLE] Prevalence of additional primary malignancies detected incidentally on PET/CT

**DOI:** 10.1590/0100-3984.2020.53.2R

**Published:** 2020

The Editorial Board of **Radiologia Brasileira** is officially communicating the Retraction for extraction of the following article:

Lee JC, Teles MS. Prevalence of additional primary malignancies detected incidentally on PET/CT. Radiol Bras. 2020;53(1):69. https://dx.doi.org/10.1590/0100-3984.2019.0097.

Reason: Duplicate publication of an article published in a previous issue of **Radiologia Brasileira**:

Lee JC, Teles MS. Prevalence of additional primary malignancies detected incidentally on PET/CT. Radiol Bras. 2019;52(5):342. https://dx.doi.org/10.1590/0100-3984.2019.0097.

Prof. Edson Marchiori

Editor-in-Chief, **Radiologia Brasileira**

